# Indents

**Published:** 1902-10-15

**Authors:** 


### INDENTS.

**☞ Brief Articles of Technical or Scientific Value will receive notice and comment. Contributions invited.**

RESIN AS A PROTECTOR OF STEEL.—Resin and lard melted together makes a good preparation for keeping steel from rusting.

CALENDULA FOR IRRITATED GUMS.—The soreness following injury to the gum from clamp, etc., may be overcome by bathing the gum with tincture of calendula.—J. E. in *Cosmos*.

TO REMOVE MATRIX FROM INLAY.—Immerse the inlay in alcohol after baking and the gold or platinum matrix can be torn off without endangering the thin margin.—Geo. Bryant, in *Dental Summary*.

GUAIACOL AS A LOCAL ANESTHETIC.—

℞ Pure guaiacol.................... grs xvj
Pure olive oil
Sterilized and neutralized ... q. s.. ad ʒiij

Sig. Inject twenty drops and wait three minutes before operating.—Dr. Narichal, July *Cosmos*, p. 784.

CELLULITIS FROM ALVEOLAR ABSCESS.—

℞. Liquor of acetate of lead . . . . fl ʒ ½

Tincture of opium . . . . . . fl ʒ 2

Distilled water . . . . . . . ℥ vj

Sig. Saturate a compress and apply hot to the inflamed structure.—*Cosmos*.

MALARIAL PERIOSTITIS.—

℞. Acetanelid,
Quinine.......... āā grs. xxjv
Sulphate of codein........ gr. j

Sig.—Make into twelve powders or capsules and take one every two or three hours.—J. R. Megrow, *Western Journal*.

